# Quantum Dots Mediated Crystallization Enhancement in Two-Step Processed Perovskite Solar Cells

**DOI:** 10.1007/s40820-025-01677-5

**Published:** 2025-02-27

**Authors:** Heng Liu, Geyu Jin, Jiantao Wang, Weihai Zhang, Long Qing, Yao Zhang, Qiongqiong Lu, Pengfei Yue, Guoshang Zhang, Jing Wei, Hongbo Li, Hsing-Lin Wang

**Affiliations:** 1 Henan Key Laboratory of Advanced Conductor Materials, Institute of Materials, Henan Academy of Sciences, Zhengzhou, 450046 People’s Republic of China; 2 Department of Materials Science and Engineering, Southern University of Science and Technology, Shenzhen, 518055 People’s Republic of China; 3 Beijing Key Laboratory of Construction-Tailorable Advanced Functional Materials and Green Applications, Experimental Center of Advanced Materials, School of Materials Science and Engineering, Beijing Institute of Technology, Beijing, 100081 People’s Republic of China; 4 College of New Energy, Ningbo University of Technology, Ningbo, 315336 People’s Republic of China

**Keywords:** Quantum dots, Perovskite solar cells, Two-step, Crystallization, Efficiency, Stability

## Abstract

Hybrid organic–inorganic lead halide perovskites have emerged as a promising material for high-efficiency solar cells, yet challenges related to crystallization and defects limit their performance and stability. This study investigates the use of perovskite quantum dots (QDs) as crystallization seeds to enhance the quality of FAPbI_3_ perovskite films and improve the performance of perovskite solar cells (PSCs). We demonstrate that CsPbI_3_ and CsPbBr_3_ QDs effectively guide the crystallization process, leading to the formation of larger crystals with preferential orientations, particularly the (001) and (002) planes, which are associated with reduced defect densities. This seed-mediated growth strategy resulted in PSCs with power conversion efficiencies (PCEs) of 24.75% and 24.11%, respectively, compared to the baseline efficiency of 22.05% for control devices. Furthermore, devices incorporating QD-treated perovskite films exhibited remarkable stability, maintaining over 80% of their initial PCE after 1000 h of simulated sunlight exposure, a significant improvement over the control. Detailed optoelectronic characterization revealed reduced non-radiative recombination and enhanced charge transport in QD-treated devices. These findings highlight the potential of QDs as a powerful tool to improve perovskite crystallization, facet orientation, and overall device performance, offering a promising route to enhance both efficiency and stability in PSCs.

**Supplementary Information:**

The online version contains supplementary material available at 10.1007/s40820-025-01677-5.

## Highlights

- The incorporation of quantum dots (QDs) as crystallization seeds results in the growth of larger perovskite crystals with reduced defect densities and preferential orientations along the (001) and (002) planes, significantly improving the film morphology.
- The QD-seeded films exhibit reduced non-radiative recombination and enhanced charge transport, as confirmed by steady-state and time-resolved photoluminescence, transient photovoltage measurements, and electrochemical impedance spectroscopy.
- Devices fabricated with QD-treated films achieve a remarkable power conversion efficiency (PCE) of 24.75% and exhibit exceptional long-term stability under simulated sunlight exposure, retaining 80% of their PCE after 1000 h of continuous illumination.

**Supplementary Information:**

The online version contains supplementary material available at 10.1007/s40820-025-01677-5.

## Graphical abstract

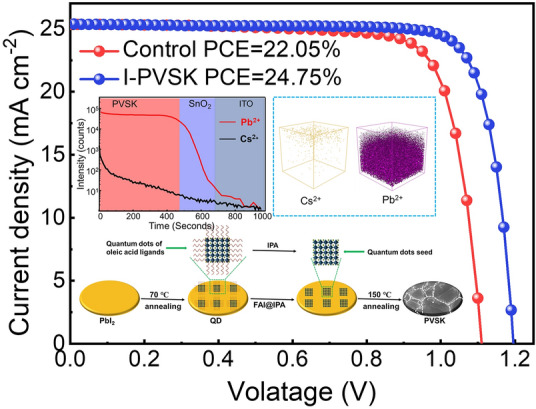

## Introduction

Hybrid organic–inorganic lead halide perovskites have garnered significant attention due to their exceptional optoelectronic properties, including tunable bandgaps, high absorption coefficients, and long carrier diffusion lengths [1–4]. Perovskite solar cells (PSCs) have seen remarkable progress in power conversion efficiency (PCE), with a certified record of 26.7% [5]. However, economically viable, solution-based fabrication of efficient PSCs remains challenging [6]. Key issues include controlling solvent evaporation and perovskite crystallization, which often results in defects and vacancies at surfaces and grain boundaries of polycrystalline perovskite films [7–9]. These defects can promote non-radiative recombination, leading to performance degradation and reduced stability in PSCs [10, 11].

To enhance perovskite film quality, understanding and controlling the crystallization process to minimize defects is crucial for improving both efficiency and device longevity [12–14]. Strategies such as additive engineering [15], solvent engineering [16], intermediate phase modulation [17], and annealing environment control [18] have been explored. While these approaches have improved PSC performance and stability, developing new methods to fundamentally control perovskite crystal growth remains a major challenge. In two-step processed PSCs, although reproducibility has been high, issues with incomplete conversion of lead salts to perovskite still persist [19, 20]. Epitaxial growth is a promising technique for directing crystal growth and producing high-quality, low-defect-density films [21]. The performance of PSCs, including carrier lifetime, open-circuit voltage deficits, and device hysteresis, is strongly influenced by defects in the perovskite (111) planes [22]. Addressing these defects is crucial to improve PSC efficiency. For example, Cao et al. demonstrated that two-dimensional WS_2_ flakes could serve as substrates for van der Waals epitaxial growth of hybrid perovskite thin films, promoting preferential growth along the (001) orientation and reducing defect density [23]. Tang et al. discovered that the perovskite (111) planes had the highest defect density, whereas the (100) planes exhibited the lowest [24]. Luo et al. enhanced the quality of perovskite films by utilizing a highly oriented (BDA)PbI_4_ perovskite template to seed the epitaxial growth of three-dimensional perovskite films along the (001) plane. This approach led to an improvement in power conversion efficiency (PCE) from 21.03% to 23.95% and also increased device stability [25]. These studies underscore the importance of crystal orientation in improving the optoelectronic properties and performance of PSCs, highlighting the need for strategies to promote favorable orientations while suppressing detrimental ones.

In recent years, perovskite quantum dots (QDs) have emerged as a promising tool for improving perovskite film quality, reducing defects, and enhancing device performance [26]. QDs such as CsPbX_3_ (*X* = Cl, Br, I) offer high carrier mobility, chemical stability, and lattice compatibility with organic–inorganic perovskites, making them effective in seeding the crystallization and growth of perovskite films [27]. For instance, Hu et al. demonstrated that incorporating CsPbBr_3_ QDs into perovskite thin films, followed by thermal annealing, suppressed phase segregation, reduced defects, and improved film stability [28]. Zhuang et al. integrated Ln^3+^-doped CsPbBrCl_2_ QDs into PSCs, achieving defect passivation in the MAPbI_3_ layers and enhancing device stability and performance by optimizing the work function and band alignment [29]. Despite these advances, using perovskite QDs as crystallization seeds remains underexplored. Most research has focused on utilizing QDs as photoactive layers or surface treatments, with fewer studies investigating their potential to control crystallization and facet orientation to improve PSC efficiency and stability.

In this work, we explore a seed-mediated growth approach to fabricate high-quality FAPbI_3_ films using CsPbI_3_ and CsPbBr_3_ QDs as crystallization seeds. Our results show that these QDs act as nucleation centers, guiding the crystallization process toward the formation of larger crystals with preferential orientations, particularly the (001) and (002) planes. This approach significantly reduces defects in the films and leads to PSCs with improved PCEs of 24.75% and 24.11% for CsPbI_3_ and CsPbBr_3_ QDs, respectively, compared to the baseline PCE of 22.05%. Furthermore, devices based on these quantum-dot-seeded perovskite films maintain 87.6% and 83.8% of their initial PCE after 1000 h of simulated AM 1.5G sunlight exposure and maximum power point tracking, demonstrating a significant improvement in stability compared to control devices.

## Experimental and Calculation

### Materials

The ITO glass substrates were purchased from Advanced Electronic Technology Company in China. SnO_2_ was acquired from Alfa Aesar. Formamidinium Iodide (FAI), Methylammonium Bromide (MABr), Methylammonium Chloride (MACl), and Lithium-bis(trifluoromethanesulfonyl)imide (Li-TFSI) were obtained from Advanced Electronic Technology Company in China. Spiro-OMeTAD, Lead (II) iodide (PbI_2_), and 4-tert-butylpyridine were procured from Xi’an Polymer Light Technology Corp (Xi’an p-OLED). N,N-dimethylformamide (DMF), isopropyl alcohol (IPA), acetonitrile (ACN), chlorobenzene (CB), and dimethylsulfoxide (DMSO) were purchased from Sigma-Aldrich. Gold (Au, 99.99%) was obtained from commercial sources. Cesium carbonate (Cs_2_CO_3_, 99.99%), PbBr_2_ (99.99%), PbI_2_ (99.99%) were purchased from Aladdin. 1-octadecene (ODE, 90%), oleic acid (OA, 90%), oleylamine (OAm, 80%–90%) were purchased from Alfa Aesar.

### Solution Preparation and Device Fabrication

#### Solution Preparation

The 12 wt% SnO_2_ colloidal solution was diluted in deionized water (1:3, v:v) and stirred for 10 min at room temperature, followed by filtration using a syringe and an aqueous filter. For the preparation of the PbI_2_ precursor solution, 691.5 mg of PbI_2_ powder was dissolved in 1 mL of DMF/DMSO (900:100) and stirred overnight at 70 °C. To prepare the organic amine salt solution, an isopropyl alcohol (IPA) solution containing organic salts (with a mass ratio of FAI:MACl of 90:15 mg) was stirred at 70 °C for 30 min. The preparation of the Spiro-OMeTAD HTL solution included 72.3 mg of Spiro-OMeTAD, 28.8 μL of 4-tertbutylpyridine, 17.5 μL of lithium-bis (trifluoromethanesulfonyl) imide (Li-TFSI) solution (520 mg Li-TFSI in 1 mL acetonitrile), and 1 mL of chlorobenzene. Cs_2_CO_3_ (0.2 g), ODE (10 mL), OA (0.7 mL) were loaded into a 50 mL flask and degassed for 1 h at 100 °C and then heated to 150 °C under N_2_ atmosphere until the Cs_2_CO_3_ was completely dissolved.

#### Quantum Dots Preparation

CsPbBr_3_ QDs and CsPbI_3_ QDs were synthesized through a reported method with minor modifications. PbX_2_ (0.43 mmol) such as PbBr_2_ (0.157 g) or PbI_2_ (0.2 g), ODE (10 mL), OA (1 mL) and OAm (1 mL) were loaded into a 50 mL flask and degassed for 1 h at 100 °C and then heated to 150 °C under N_2_ atmosphere to form a clear solution. The temperature was increased to 170 °C, followed by the quick injection of 0.8 mL Cs-OA solution. 5 s after the injection, the reaction was stopped with an ice bath. The ethyl acetate was added to the crude solution (Ethyl acetate: crude solution = 1:1 by volume) to precipitate QDs, then the mixture solution was centrifugation at 7000 rpm for 3 min. The collected CsPbX_3_ QDs were dispersed in toluene to form a solution with 15 mg mL^−1^ concentration.

#### Device Fabrication

The ITO glass substrates were initially cleaned with a detergent, followed by ultrasonic cleaning in deionized water, acetone, and isopropanol for 30 min each. The ITO glass was dried with nitrogen gas before undergoing a 5-min plasma treatment. Subsequently, SnO_2_ was spin-coated onto the substrates at a speed of 3500 rpm to serve as the electron transport layer, followed by thermal annealing at 150 °C for 30 min on a hot plate. After cooling to room temperature, the substrates were transferred to a nitrogen-filled glovebox. A 1.5 M PbI_2_ solution (in anhydrous DMF: DMSO at a volume ratio of 9:1) was then spin-coated onto the SnO_2_ layer at 1700 rpm, annealed at 70 °C for 1 min, and allowed to cool for 10 min. Quantum dot solution, at a concentration of 3 mg mL^-1^, was then spin-coated on the PbI_2_ layer, followed by a 70 °C anneal for 1 min. Afterward, a FAI:MACl solution (90:15 mg in 1 mL IPA) was spin-coated on the PbI_2_ layer at 1800 rpm for 30 s, after which the substrates were transferred to an air atmosphere glovebox (RH 30%–40%) for thermal annealing at 150 °C for 10 min on a hot plate. Post-annealing, the substrates were cooled to room temperature in a nitrogen-filled glovebox. Next, a Spiro-OMeTAD solution was spin-coated onto the prepared perovskite film at 4000 rpm for 30 s. Lastly, a gold electrode approximately 80 nm thick was thermally evaporated under high vacuum through a mask.

### Characterizations

The crystal structure and phases of the perovskite were characterized using a Bruker Advanced D8 X-ray diffractometer under Cu Kα (*λ* = 0.154 nm) radiation. The absorbance spectra of the perovskite film were obtained using a UV–Vis spectrophotometer (Agilent Cary 5000). Steady-state photoluminescence (PL) spectra were recorded on a Shimadzu RF-5301pc, while time-resolved photoluminescence spectra were acquired using a picosecond pulsed laser excitation with a 1 MHz repetition rate through a photoluminescence system (Fluo-Time 300). The morphology of the film was investigated using a scanning electron microscope (SEM; TESCAN MIRA3). The surface potential of the perovskite film was measured using an atomic force microscope (AFM; Asylum Research MFP-3D-Stand Alone). X-ray photoelectron spectroscopy (XPS) was performed on a Thermo K-Alpha + spectrometer equipped with a monochromatic Al Kα X-ray source (1486.6 eV) operating at 100 W power. The samples were analyzed under vacuum (*P *< 10^–8^ mbar), calibrated through 150 eV (survey scan) or 50 eV (high-resolution scan) energy pass, with a binding energy of C 1*s* at 284.8 eV for calibration. Ultraviolet photoelectron spectroscopy (UPS, ESCALAB 250Xi, Thermo Fisher) measurements were carried out using a He Iα photon source (21.22 eV). Current–voltage (*J-V*) curves for fabricated devices were obtained by collecting data under forward and reverse scans with 30 mV intervals and a 10 ms delay time, under AM 1.5 G illumination (100 mW cm^−2^) using a source meter (Keysight B2901A) and a solar simulator (Enlitech SS-F5-3A). The external quantum efficiency (EQE) spectra were measured using an Enlitech QER-3011 quantum efficiency test system, calibrated for each wavelength’s light intensity using a Si detector before measurement. The maximum power point (MPP) output was obtained from the maximum power point current density. Electrochemical impedance spectroscopy (EIS) tests were conducted using a Princeton Applied Research P4000 + electrochemical workstation in the frequency range from 100 Hz to 1 MHz, at a bias voltage of 1 V under dark conditions with an amplitude of 10 mV.

## Results and Discussion

### Preparation and Characterization of Quantum Dots

**Fig. 1:**
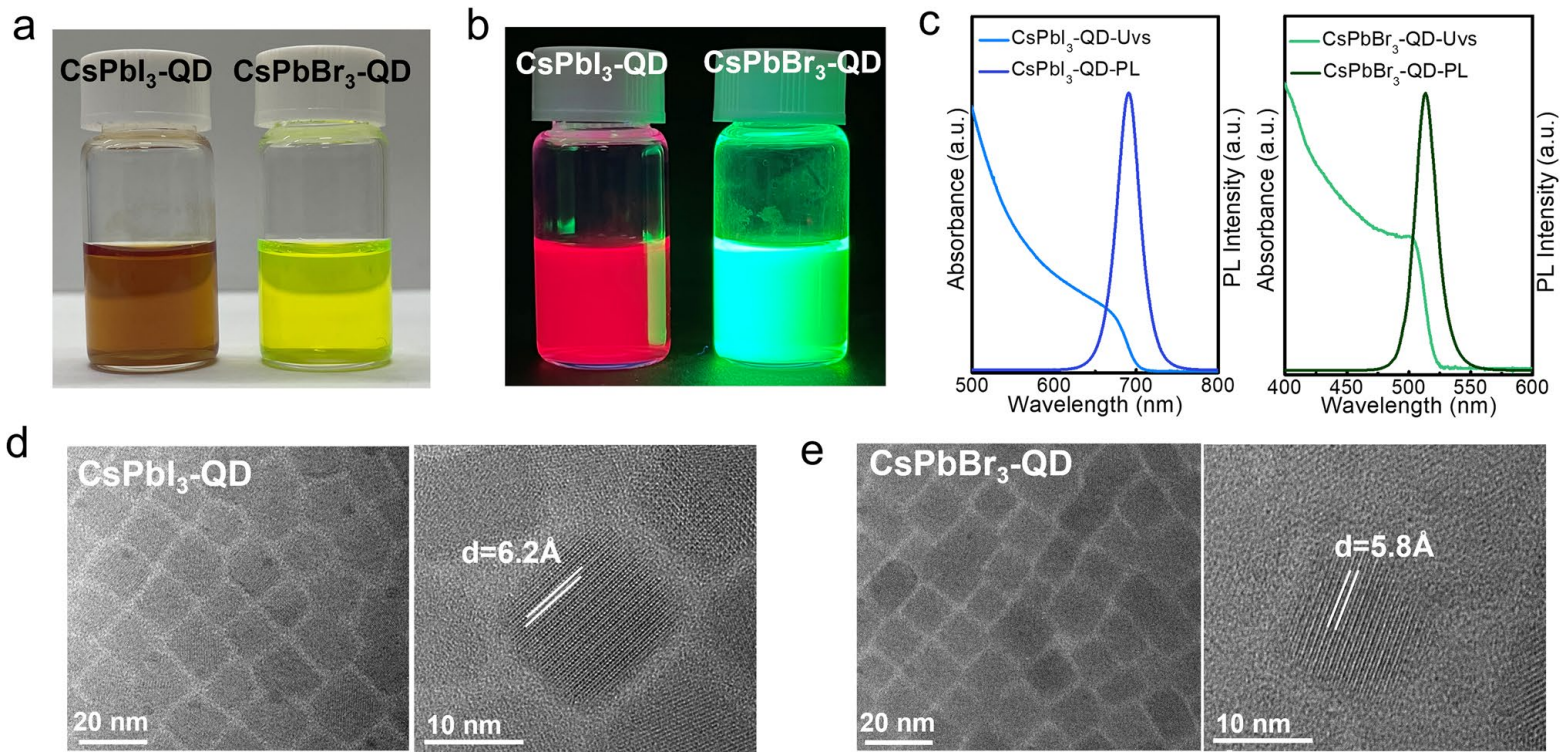
Characterizations of quantum dots. **a** QDs solution of CsPbI_3_ and CsPbBr_3_ dispersed in toluene, **b** fluorescence of CsPbI_3_ and CsPbBr_3_ solution under ultraviolet light excitation, **c** UV–Vis absorption and PL spectroscopy of CsPbI_3_ and CsPbBr_3_ QDs, **d** TEM images and high-resolutionTEM images of CsPbI_3_, **e** TEM images and high-resolutionTEM images of CsPbBr_3_ QDs

We synthesized two types of perovskite QDs: CsPbI_3_ and CsPbBr_3_, which were then dispersed in toluene to create QD solutions. As illustrated in Fig. 1a, the CsPbI_3_ solution exhibits a brown color, while the CsPbBr_3_ solution appears yellow-green. Upon excitation with ultraviolet light, the solutions transition to red and green, respectively (Fig. 1b). To investigate their optical properties, we conducted UV–visible absorption and photoluminescence (PL) spectroscopy. As shown in Fig. 1c, the absorption edges for CsPbI_3_ and CsPbBr_3_ are at 700 and 525 nm, respectively, with PL emission peaks observed at approximately 690 and 510 nm, corresponding to optical bandgaps of ~ 1.7 and ~ 2.4 eV [30]. We further examined the morphology and microstructure of the QDs using high-resolution transmission electron microscopy (HRTEM). Figure 1d, e demonstrates that both QDs are cubic in shape and uniformly distributed, with average sizes of 15.4 nm for CsPbI_3_ and 12.3 nm for CsPbBr_3_. Detailed size distribution statistics can be found in Fig. S1. High-resolution TEM images reveal distinct lattice fringes within individual QDs, with (100) interplanar spacings of 6.2 Å for CsPbI_3_ and 5.8 Å for CsPbBr_3_, consistent with previously reported values [31]. The solutions were used to fabricate perovskite films.

### Perovskite Film Morphological, Structural, and Optoelectronic Properties

**Fig. 2:**
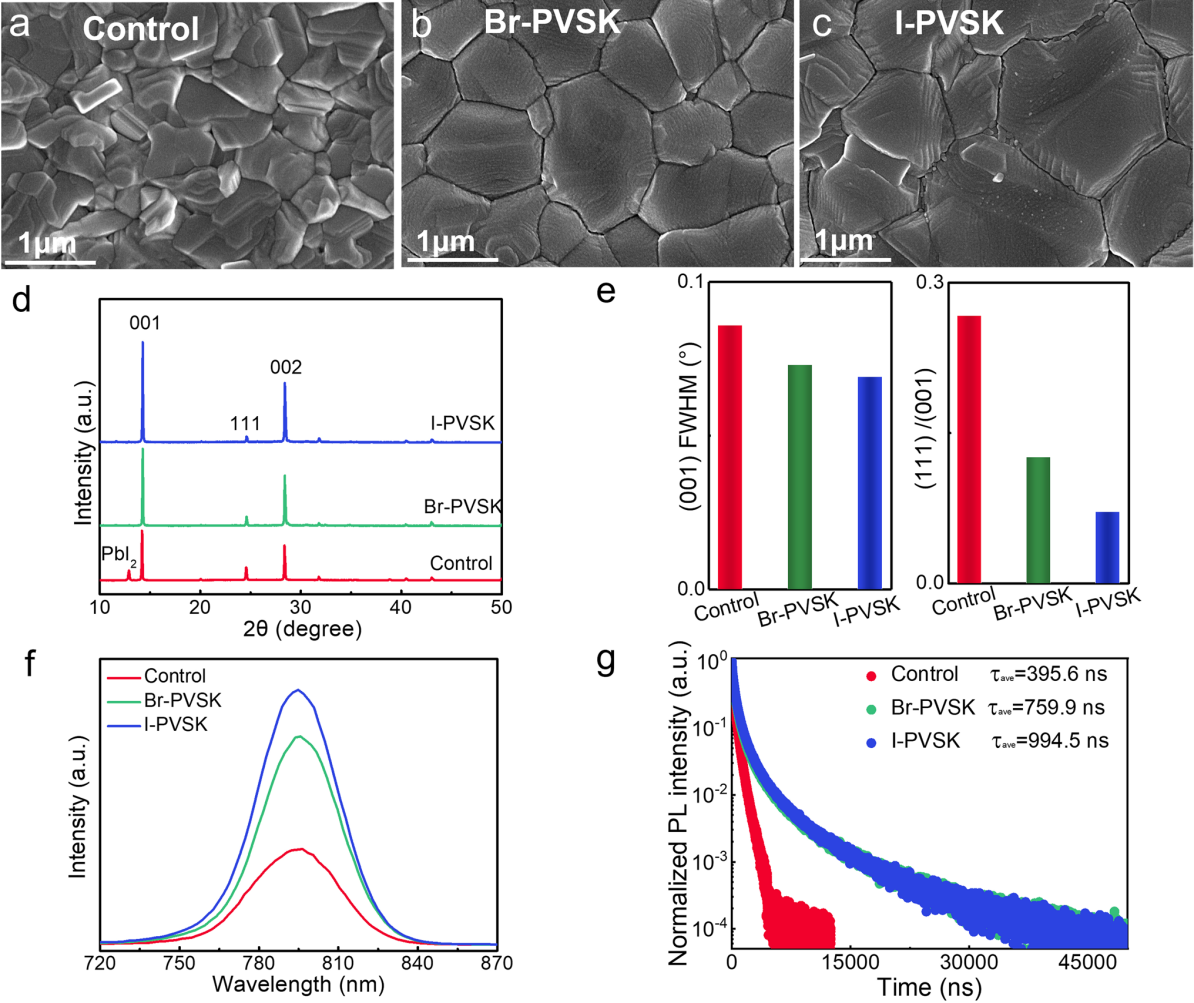
Characterizations of perovskite films. SEM surface images of **a** control, **b** Br-PVSK, and **c** I-PVSK. **d** XRD patterns of perovskite films. **e** FWHM of (001) plane and (111)/(001) plane intensity ratio derived from XRD. **f** Steady-state PL and **g** time-resolved PL of perovskite films deposited on quartzes

We prepared perovskite films using a two-step processing method [32]. Initially, lead iodide (PbI_2_) was deposited onto a glass/ITO/SnO_2_ substrate, followed by the diffusion of organic salts, including formamidinium iodide (FAI). Upon annealing, perovskite FAPbI_3_ was formed. Notably, prior to the diffusion of organic salts, we spin-coated the QDs onto the PbI_2_ layer. In subsequent experiments, the perovskite films prepared with CsPbI_3_ and CsPbBr_3_ QDs were designated as I-PVSK and Br-PVSK, respectively. We utilized scanning electron microscopy (SEM) to analyze the surface morphologies of these perovskite films. As illustrated in Fig. 2a–c, the films treated with QDs show larger grain sizes. We conducted a statistical comparison of the grain sizes for the control, Br-PVSK, and I-PVSK films (Fig. S2), measuring an average of 600 nm for the control film, 1250 nm for Br-PVSK, and 1750 nm for I-PVSK. We performed atomic force microscopy (AFM) characterization on the control, Br-PVSK and I-PVSK of perovskite films, which agreed well with the SEM results. The grain size of the perovskite films was significantly increased after quantum dot modification, as shown in Fig. S3. Furthermore, the root mean square (RMS) roughness values of these films were 36.2, 32.4, and 30.3 nm, respectively. The I-PVSK film exhibited the lowest surface roughness, which is crucial for achieving high-performance devices. Further, we have provided the cross-section SEM images of the devices based on different perovskite films, as shown in Fig. S4. It is evident that the modification with QDs has resulted in larger grain sizes and smaller grain boundaries in the Br-PVSK and I-PVSK films, which is in excellent agreement with the corresponding surface SEM images. To assess the crystallinity of the films, we conducted X-ray diffraction (XRD) analysis [33]. Figure 2d shows that all films display typical FAPbI_3_ peaks at 14.3°, 26.4°, and 28.4°, corresponding to the (001), (111), and (002) planes, respectively. The control films reveal the presence of a PbI_2_ phase at 12.9°, whereas the QD-treated films show no PbI_2_ impurities. We extracted the full width at half maximum (FWHM) values of the (001) peak intensities and compared the FWHM ratios of (111) to (001) based on their XRD patterns. As displayed in Fig. 2e, both the (001) FWHM values and the (111)/(001) ratios decrease for the control, Br-PVSK, and I-PVSK films, indicating enhanced crystallinity of the FAPbI_3_ phase after QD treatment. Further evaluation of film quality was conducted using steady-state PL and time-resolved PL (TRPL) measurements. As illustrated in Fig. 2f, the control film exhibits the lowest PL intensity, suggesting a higher level of defect-induced non-radiative recombination [34]. In contrast, the I-PVSK film displays the highest PL intensity, indicating minimal defects. The average TRPL lifetimes for the control, Br-PVSK, and I-PVSK films are 359.6, 759.9, and 994.5 ns, respectively, as summarized in Table S1 confirming a reduction in defect state densities (Fig. 2g).

### Mechanism of Quantum Dot-Mediated Crystallization for Large Grain Growth

**Fig. 3:**
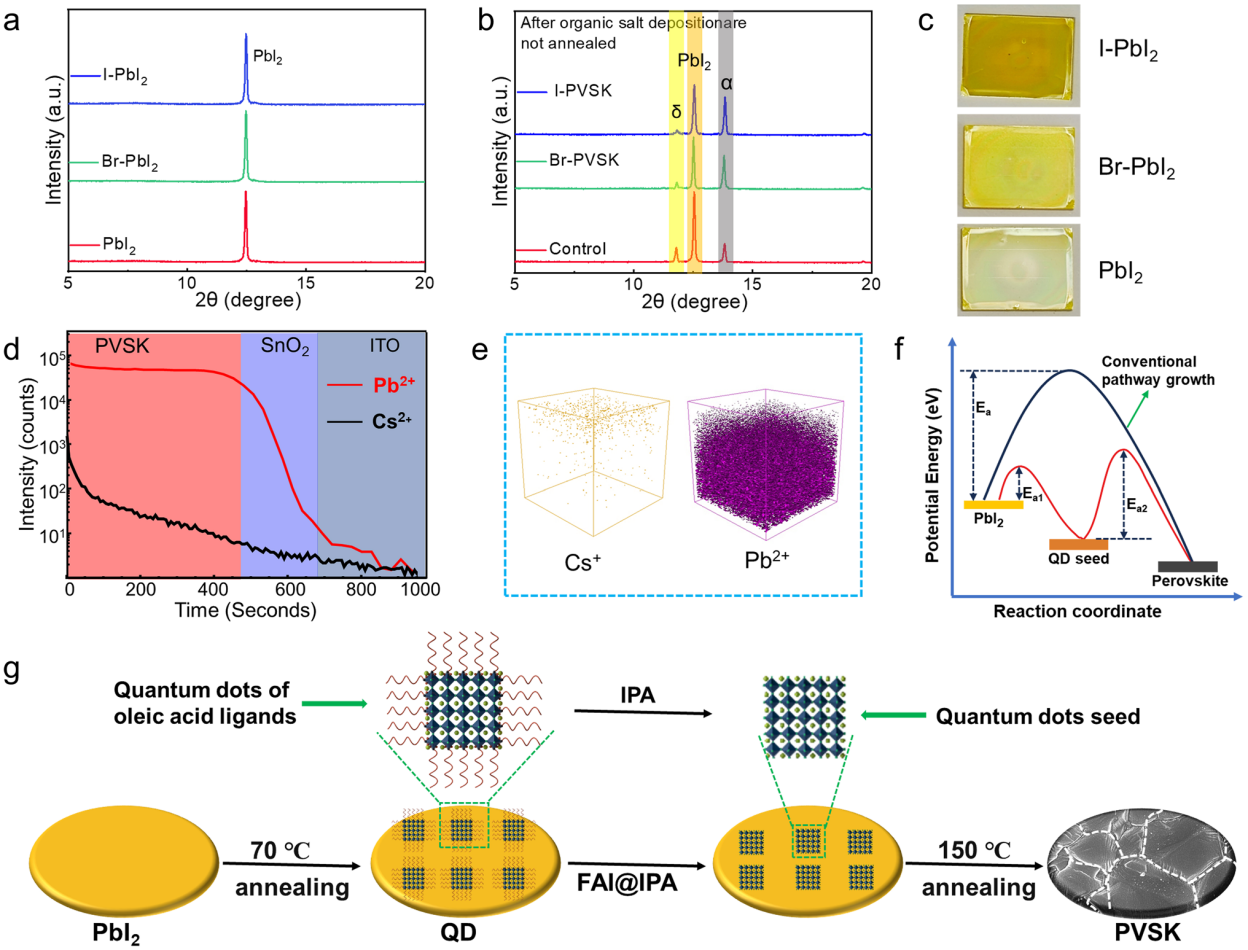
Crystallization mechanism of perovskite films. **a** XRD patterns of PbI_2_ film with and without QDs, **b** XRD patterns and **c** optical morphology of intermediate phase films in which organic cations have been deposited before film annealing, **d** ToF–SIMS of Pb^2+^ and Cs^+^ distribution in the final I-PVSK film and **e** the distribution of the ions in 3D space, **f** schematic diagram of crystallization mechanism mediated by QDs, **g** evolution of QDs during perovskite film processing

To investigate how introducing QDs influenced perovskite film formation, we examined the crystallization dynamics with and without QD treatment. Figure 3a shows the XRD patterns of PbI_2_ films before and after QD deposition. Notably, there is no significant shift in the XRD peak positions of PbI_2_. Following the spin-coating of organic cations without annealing, all films exhibit FAPbI_3_ in both δ- and α-phases (Fig. 3b) [35]. However, the δ-to-α intensity ratio decreases in Control, Br-PVSK, and I-PVSK films, indicating a shift in phase proportions. Optical images of these films are presented in Fig. 3c, where color variations are evident. The dark brown color of I-PVSK suggests enhanced conversion to α-FAPbI_3_, likely facilitated by QD diffusion and QD seed-mediated growth. To investigate the impact of organic solution processing on the morphology of deposited QDs, we conducted TEM analysis following isopropyl alcohol (IPA) washing. As shown in Fig. S5, the CsPbI_3_ and CsPbBr_3_ QDs retained their cubic morphology, consistent with the structures observed in Fig. 1d, e. To further analyze QD distribution within the final perovskite layer, we performed time-of-flight secondary ion mass spectrometry (ToF–SIMS) to map Pb^2+^ and Cs^+^ distributions in the I-PVSK film (Fig. 3d, e). The Pb^2+^ ions are uniformly distributed throughout the perovskite layer, with high intensity detected, while Cs^+^ ions originating from CsPbI_3_ QDs show reduced intensity from the surface down to the SnO_2_ layer. This suggests that QDs diffuse into the PbI_2_ layer during organic cation processing and serve as nucleation sites, enhancing perovskite conversion. A proposed mechanism is illustrated in Fig. 3f. In the conventional growth pathway without QDs, the activation energy (Ea) is high, whereas in the QD-mediated crystallization pathway, the QDs lower the conversion energy barrier [36]. To further clarify the evolution of QDs during film processing, we present a schematic in Fig. 3g. After PbI_2_ deposition, QDs capped with oleic acid ligands were introduced. During organic salt processing in IPA, some oleic acid ligands were removed, allowing the QDs to diffuse into the PbI_2_ layer as seeds. Post-annealing then enhances crystallization, with the QD seeds playing a critical role in promoting conversion within the final film.

### Performance of Perovskite Device

**Fig. 4:**
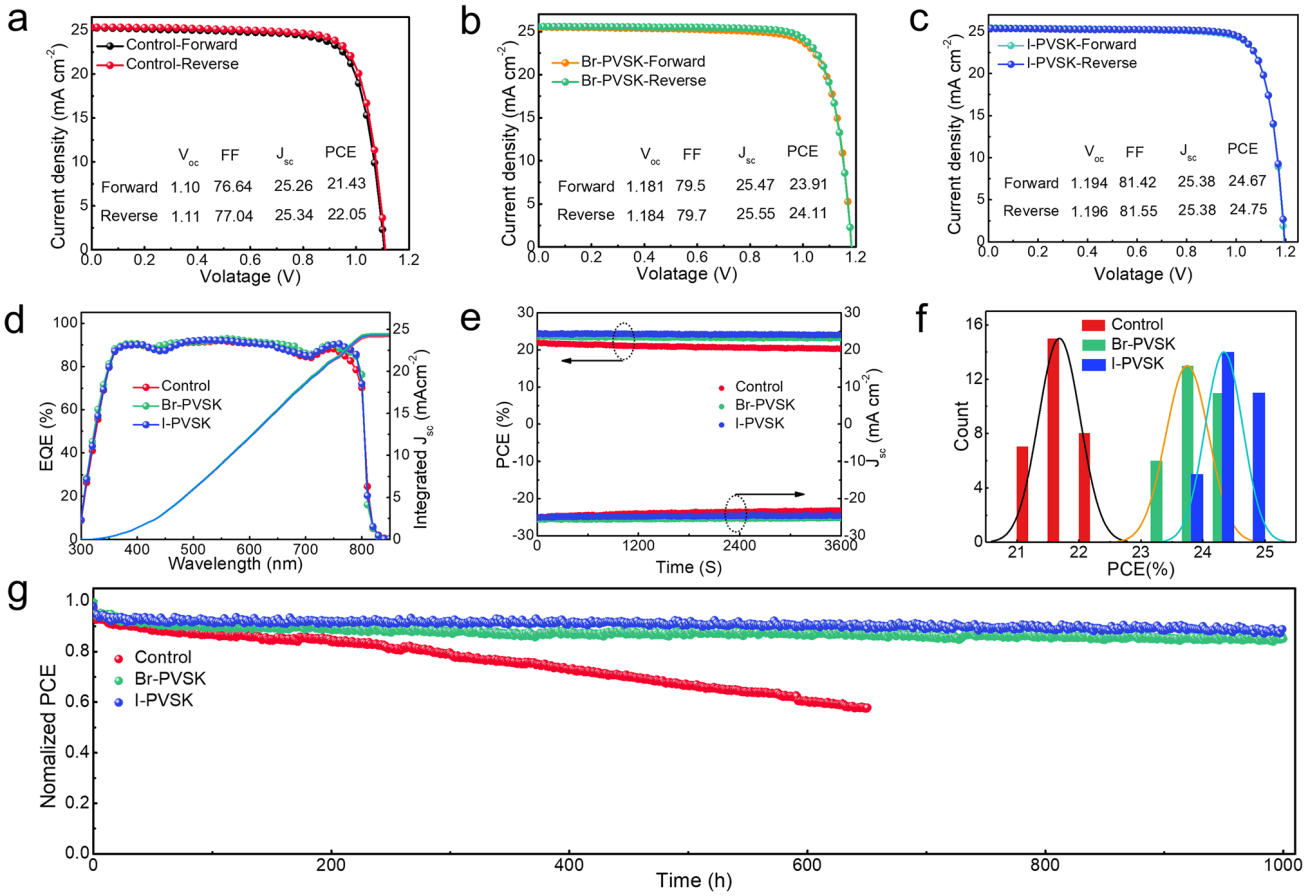
Performance measurements of PSCs. *J-V* curves of **a** Control, **b** Br-PVSK, and **c** I-PVSK PSCs, **d** external quantum efficiency (EQE) curves and integrated current densities, **e** steady power outputs, **f** device performance statistics, **g** maximum power point tracking

We fabricated PSCs with a regular configuration of ITO/SnO_2_/perovskite with or without QD treatment/Spiro-OMeTAD/Au. The current density–voltage (*J-V*) curves are presented in Fig. 4a–c. Their detailed photovoltaic parameters are listed in Table S3. The control device achieved a PCE of 22.05% under reverse scan measurements, with an open circuit voltage (*V*_oc_) of 1.110 V, a short circuit current density (*J*_sc_) of 25.34 mA cm^−2^, and a fill factor (FF) of 77.04%, which is consistent with previous reports [37]. Following the treatment with CsPbBr_3_ QDs, the device PCE increased to 24.11%, with a *V*_oc_ of 1.184 V, a *J*_sc_ of 25.55 mA cm^−2^, and an *FF* of 77.04%. The treatment with CsPbI_3_ QDs further enhanced the device PCE to 24.75%, with a *V*_oc_ of 1.196 V, a *J*_sc_ of 25.38 mA cm^−2^, and an FF of 81.55%. The noticeable enhancement in *V*_oc_ under reverse scan conditions indicates a reduction in non-radiative recombination following the introduction of QDs. To confirm the current density results, we performed external quantum efficiency (EQE) measurements. As shown in Fig. 4d, QD treatment slightly improved the EQE in the infrared absorption region. The integrated *J*_sc_ values derived from the EQE curves were 24.2, 24.5, and 24.3 mA cm^−2^ for the control, Br-PVSK, and I-PVSK devices, respectively. We also measured the steady power output (SPO) of the three devices to assess their operating efficiencies accurately. As displayed in Fig. 4e, the PCEs under steady operation were 22.2%, 24.1%, and 24.4% for the control, Br-PVSK, and I-PVSK devices, respectively, which aligns well with the *J-V* results. We investigated the reproducibility of the devices by conducting PCE statistics on 30 devices under each condition. As shown in Fig. 4f, the average PCEs with standard deviations for the control, Br-PVSK, and I-PVSK devices were 21.7 ± 0.33%, 23.7 ± 0.34%, and 24.3 ± 0.29%, respectively. These statistical results are consistent with our *J-V* measurements, indicating a solid trend of efficiency enhancement through incorporating QDs, with CsPbI_3_ QDs demonstrating greater efficacy. To demonstrate the positive effect of QDs on device stability, high temperature and humidity conditions for the unencapsulated devices were investigated, and the corresponding PCE decay was presented in Fig. S6. Clearly, under the same conditions, quantum dot devices based on thin films exhibit superior stability compared to control devices, with CsPbI_3_ quantum dot-modified devices demonstrating the greatest stability. Notably, as illustrated in Fig. S6c, there is a significant enhancement in the stability against high temperature and humidity. The I-PVSK film-based devices retained over 80% of their original PCE after being exposed to air for 20 days, whereas the control devices rapidly dropped to only 35% of their initial value. These results indicate that quantum dot devices demonstrate better stability than the control devices under high-temperature and high-humidity conditions. This is primarily attributed to the quantum dot modification, which improves film quality and reduces defect density, thereby enhancing the stability of the perovskite thin films. Additionally, we studied the stability of the devices by tracking their maximum power point (MPP) efficiencies under one sun-intensity illumination. The MPP tracking stability of unencapsulated devices was monitored in a nitrogen-filled glove box. As shown in Fig. 4g, after continuous exposure for 1000 h, the I-PVSK and Br-PVSK devices retained approximately 87.6% and 83.8% of their initial PCE, respectively, while the control devices dropped to below 50% after 650 h.

**Fig. 5:**
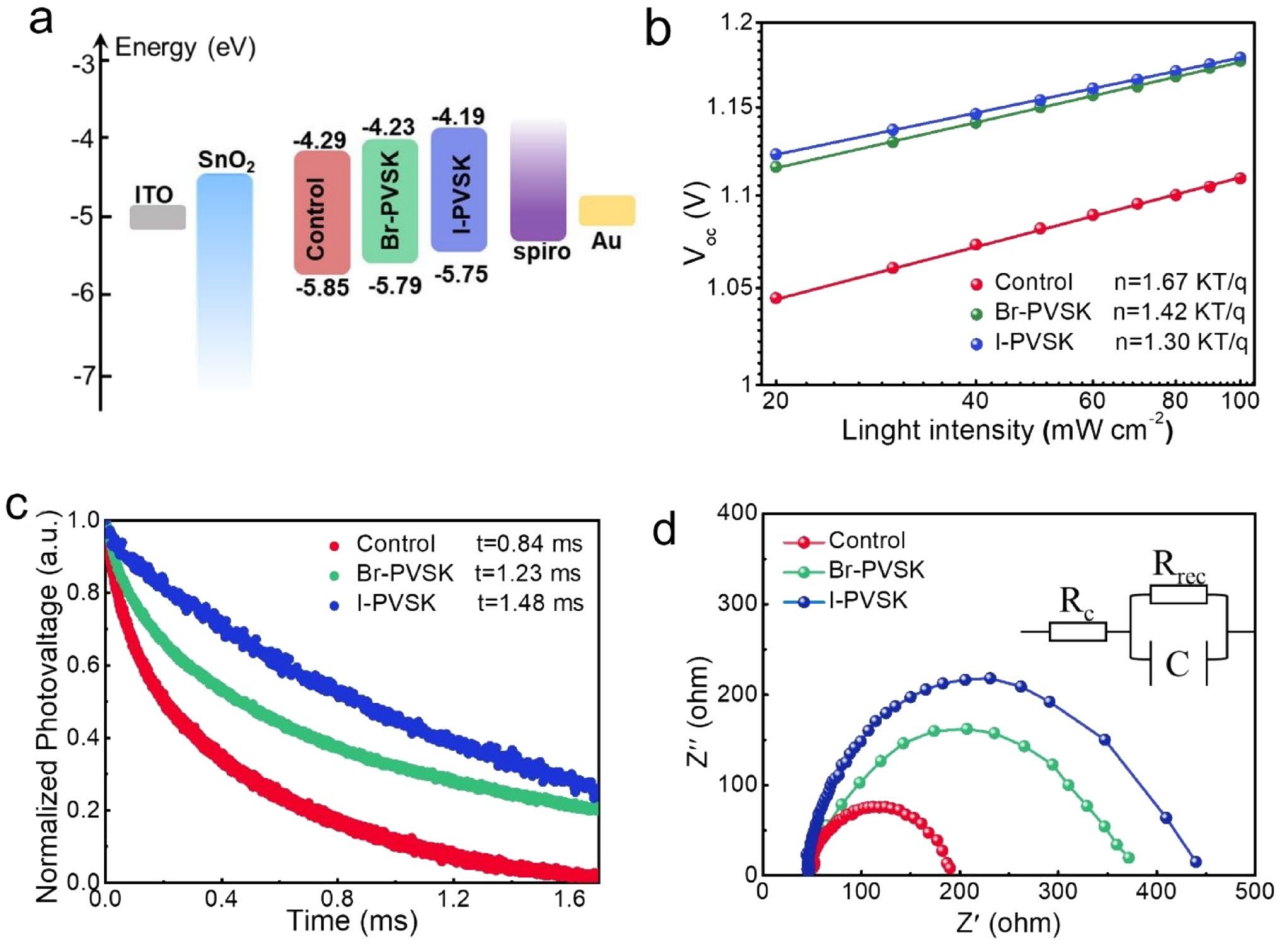
Device characterizations. **a** Energy level alignment, **b** open circuit voltage changes as light intensity, **c** transient photovoltage curves, **d** Nyquist plots of electrochemical impedance spectroscopy

To investigate the underlying mechanisms behind performance enhancement, we conducted comprehensive optoelectronic characterizations. Ultraviolet photoelectron spectroscopy (UPS) was used to analyze the electronic structure of the thin films [38]. Figure S7 shows the secondary electron cutoff energy (*E*_cut-off_) and onset energy (*E*_onset_) for the control, Br-PVSK, and I-PVSK films. Table S4 summarizes the conduction band minimum, valence band maximum, and work function of the films, derived from the optical bandgap calculated from UV–vis absorption spectra (Fig. S8). Table S4 shows that the work functions for the control, Br-PVSK, and I-PVSK films decreased from 4.57 to 4.54 eV, and further to 4.52 eV, respectively. The energy level diagram of the perovskite, derived from UPS results, is illustrated in Fig. S9. The decrease in work function results in an upward shift of both the valence and conduction bands, thereby reducing the energy level difference between the perovskite layer and the hole transport layer, Spiro-OMeTAD [39, 40]. This reduction in work function facilitates charge carrier extraction and transport between the perovskite and the hole transport layer (HTL) [7]. We performed Kelvin probe force microscopy (KPFM) measurements in a dark environment to investigate the local surface potential of the films (Fig. S10). The contact potential difference (CPD) represents the potential difference between the film’s surface and the probe, described by the equation: $\text{CPD}=\frac{{W}_{\text{tip}}-{W}_{\text{sample}}}{e}$, where *W*_tip_ and *W*_sample_ denote the work functions (WF) of the probe and the sample, respectively [41]. By subtracting the measured CPD from the work function of the probe, the work function of the sample can be derived. The data presented in the insets of Fig. S10a–c reveal that the average CPD for the control, Br-PVSK and I-PVSK films increased from 400 to 490 mV, and 515 mV, respectively. The highest CPD value recorded for the I-PVSK film reflects the lowest work function, which enhances charge extraction efficiency in the corresponding device. This observation aligns with the findings from ultraviolet photoelectron spectroscopy (UPS), indicating that a lower work function is beneficial for charge extraction and transport. Figure 5a displays the energy level diagrams for perovskite devices incorporating the different films, showing an upward shift in the valence band edge due to introducing QDs. This change reduces the energy gap between the perovskite and the HTL, which promotes more efficient hole collection and enhances device performance. In order to investigate the impact of QDs modifications on hole extraction, we characterized the Glass/PVSK/Spiro-OMeTAD, Glass/Br-PVSK/Spiro-OMeTAD and Glass/I-PVSK/Spiro-OMeTAD films using PL and TRPL measurements. As shown in Fig. S11a, Glass/Br-PVSK/Spiro-OMeTAD and Glass/I-PVSK/Spiro-OMeTAD film shows a faster PL quench than that of Glass/PVSK/Spiro-OMeTAD film, indicating that QDs modification can enhance the hole extraction efficiency. The mechanism behind can be attributed to the reduced work function as supported by the KPFM and UPS results. TRPL was further conducted to determine the charge carrier lifetime of the Glass/PVSK/Spiro-OMeTAD, Glass/Br-PVSK/Spiro-OMeTAD and Glass/I-PVSK/Spiro-OMeTAD films, and the results were fitted by a bi-exponential decay function with detailed parameters summarized in Table S2. As shown in Fig. S11b, the average carrier lifetime of the Glass/PVSK/Spiro-OMeTAD, Glass/Br-PVSK/Spiro-OMeTAD and Glass/I-PVSK/Spiro-OMeTAD films are 271.3, 201.2, and 158.8 ns, respectively. The shortest carrier lifetime in the Glass/I-PVSK/Spiro-OMeTAD film agrees well with its fastest PL quench, which further suggested that CsPbI_3_ QDs modification can promote charge carrier extraction. To further examine carrier recombination and extraction kinetics, we analyzed the dependence of open-circuit voltage (*V*_oc_) on light intensity [42]. The plot of *V*_oc_ versus light intensity (Fig. 5b) shows a logarithmic relationship, where the calculated slopes are 1.67, 1.42, and 1.3 kT q^−1^ for the control, Br-PVSK, and I-PVSK devices, respectively (with k as the Boltzmann constant, T as the absolute temperature, and q as the elementary charge). The reduction in slope for QD-treated devices indicates a significant suppression of trap-assisted recombination due to enhanced perovskite crystallinity, effectively reducing *V*_oc_ losses. The smallest slope for the I-PVSK device, in particular, suggests the lowest level of trap-assisted recombination, consistent with its high *V*_oc_ of 1.196 V. Further, the space-charge-limited current (SCLC) technique was employed to estimate the defect density of the perovskite films. Figure S12a–c presents the dark current–voltage (*J-V*) characteristics of the electron-only devices, from which the trap-filled limit voltages (*V*_TFL_) for the control, Br-PVSK, and I-PVSK devices were determined to be 0.68, 0.48, and 0.43 V, respectively. It is widely accepted that V_TFL_ can be used to estimate the trap state density (*N*_*t*_) of the films using the following equation: ${N}_{t}=\frac{2{\varepsilon }_{0}\varepsilon {V}_{\text{TFL}}}{e{L}^{2}}$, where *ɛ* is the relative permittivity of the perovskite, with a value of 62.23, *ɛ*_0_ is the vacuum permittivity, e is the elementary charge, and *L* is the thickness of the perovskite film[43], approximately 800 nm, as shown in Fig S4. Accordingly, the trap state densities of the control, Br-PVSK, and I-PVSK films were calculated to be 1.41 × 10^16^, 9.97 × 10^15^, and 8.92 × 10^15^ cm⁻^3^, respectively. The lowest defect density of the I-PVSK film is closely associated with its improved crystallinity and the passivation of surface defects. Transient photovoltage (TPV) measurements (Fig. 5c) reveal decay times of 0.84, 1.23, and 1.48 ms for the control, Br-PVSK, and I-PVSK devices, respectively, confirming that QD treatment suppresses carrier recombination [44]. Electrochemical impedance spectroscopy (EIS) measurements performed under dark conditions provide further insights into carrier transport. The Nyquist plots were measured under dark conditions at room temperature with a bias voltage of 1 V. Figure 5d presents the Nyquist plots of the devices, and the inset illustrates the corresponding equivalent circuit model, which includes series capacitance (C), charge transport resistance (*R*_c_), and recombination resistance (*R*_rec_). Generally, the high-frequency component can be attributed to *R*_c_, while the low-frequency component is associated with *R*_rec_ [45]. In the high-frequency region, the values of *R*_c_ for the control group, Br-PVSK, and I-PVSK devices were 49, 40, and 35 Ω, respectively. The lowest *R*_c_ value observed in the I-PVSK device can be attributed to better energy level alignment, which significantly enhances charge carrier extraction efficiency. In the low-frequency region, the *R*_rec_ values for the control, Br-PVSK, and I-PVSK devices were fitted to 187, 374, and 438 Ω, respectively. The corresponding recombination resistance (*R*_rec_) values show higher *R*_rec_ in QD-treated devices compared to controls, indicating reduced charge recombination and increased free carrier density [46]. The largest semicircle and highest *R*_rec_ in the Nyquist plot for the I-PVSK device highlight its superior suppression of charge recombination.

## Conclusions

In this study, we demonstrate the successful application of perovskite QDs as crystallization seeds to enhance the quality of FAPbI_3_ perovskite films, thereby improving the performance and stability of perovskite solar cells (PSCs). By incorporating CsPbI_3_ and CsPbBr_3_ QDs, we effectively promoted nucleation, leading to larger crystallites with preferential orientations, notably the (001) and (002) planes, while reducing the (111) orientations associated with higher defect densities. The QD-mediated growth approach resulted in perovskite films with enhanced crystallinity, reduced defect-induced non-radiative recombination, and improved optoelectronic properties. As a result, PSCs fabricated with QD-seeded perovskite films exhibited significant improvements in PCE, reaching 24.75% and 24.11% for CsPbI_3_ and CsPbBr_3_ QDs-based PSCs, respectively, compared to a baseline of 22.05% for the control devices. These devices also demonstrated remarkable stability, maintaining over 80% of their initial PCE after 1000 h of simulated sunlight exposure, highlighting the potential of QDs to enhance device longevity. Additionally, optoelectronic characterization revealed reduced carrier recombination and enhanced charge extraction, further corroborating the effectiveness of the QD treatment. These findings underscore the promise of QDs as a powerful tool for tailoring perovskite crystallization and facet orientation, offering a route to improve both the efficiency and stability of PSCs.

## Supplementary Information

Below is the link to the electronic supplementary material.

Supplementary file1 (DOCX 2554 KB)
