# Supplementary material for: Quantum Dots Mediated Crystallization Enhancement in Two-Step Processed Perovskite Solar Cells
Source: Nanomicro Lett. 2025 Feb 27;17:169. doi: 10.1007/s40820-025-01677-5 (PMC11865417; doi:10.1007/s40820-025-01677-5)
Supplement: Supplementary file 1 — Supplementary file1 (DOCX 2554 KB) [file 40820_2025_1677_MOESM1_ESM.docx]

Supporting Information for

**Quantum Dots Mediated Crystallization Enhancement in Two-Step Processed Perovskite Solar Cells**

Heng Liu^1,2, #^, Geyu Jin^3, #^, Jiantao Wang^2^*, Weihai Zhang^4^, Long Qing^2^, Yao Zhang^3^, Qiongqiong Lu^1^, Pengfei Yue^1^, Guoshang Zhang^1^, Jing Wei^3^*, Hongbo Li^3^*, Hsing-Lin Wang^2^*

^1^Henan Key Laboratory of Advanced Conductor Materials, Institute of Materials, Henan Academy of Sciences, Zhengzhou 450046, P. R. China

^2^Department of Materials Science and Engineering, Southern University of Science and Technology, Shenzhen 518055, Guangdong, P. R. China

^3^Beijing Key Laboratory of Construction-Tailorable Advanced Functional Materials and Green Applications, Experimental Center of Advanced Materials, School of Materials Science and Engineering, Beijing Institute of Technology, Beijing 100081, P. R. China

^4^College of New Energy, Ningbo University of Technology, Ningbo 315336, P. R. China

^#^Heng Liu and Geyu Jin contributed equally to this work.

*Corresponding authors. [jwangdz@connect.ust.hk](mailto:jwangdz@connect.ust.hk) (Jiantao Wang); [weijing@bit.edu.cn](mailto:weijing@bit.edu.cn) (Jing Wei); [hongbo.li@bit.edu.cn](mailto:hongbo.li@bit.edu.cn) (Hongbo Li); [wangxl3@sustech.edu.cn](mailto:wangxl3@sustech.edu.cn) (Hsing-Lin Wang)

S1 Experimental Section

***Materials:*** The ITO glass substrates were purchased from Advanced Electronic Technology Company in China. SnO_2_ was acquired from Alfa Aesar. Formamidinium Iodide (FAI), Methylammonium Bromide (MABr), Methylammonium Chloride (MACl), and Lithium-bis(trifluoromethanesulfonyl)imide (Li-TFSI) were obtained from Advanced Electronic Technology Company in China. Spiro-OMeTAD, Lead (II) iodide (PbI_2_), and 4-tert-butylpyridine were procured from Xi'an Polymer Light Technology Corp (Xi'an p-OLED). N,N-dimethylformamide (DMF), isopropyl alcohol (IPA), acetonitrile (ACN), chlorobenzene (CB), and dimethylsulfoxide (DMSO) were purchased from Sigma-Aldrich. Gold (Au, 99.99%) was obtained from commercial sources. Cesium carbonate (Cs_2_CO_3_, 99.99%), PbBr_2_ (99.99%), PbI_2_ (99.99%) were purchased from Aladdin. 1-octadecene (ODE, 90%), oleic acid (OA, 90%), oleylamine (OAm, 80–90%) were purchased from Alfa Aesar.

***Solution Preparation:*** The 12 wt% SnO_2_ colloidal solution was diluted in deionized water (1:3, v:v) and stirred for 10 minutes at room temperature, followed by filtration using a syringe and an aqueous filter. For the preparation of the PbI_2_ precursor solution, 691.5 mg of PbI_2_ powder was dissolved in 1 mL of DMF/DMSO (900:100) and stirred overnight at 70 °C. To prepare the organic amine salt solution, an isopropyl alcohol (IPA) solution containing organic salts (with a mass ratio of FAI:MACl of 90 mg:15 mg) was stirred at 70 °C for 30 minutes. The preparation of the Spiro-OMeTAD HTL solution included 72.3 mg of Spiro-OMeTAD, 28.8 μL of 4-tertbutylpyridine, 17.5 μL of lithium-bis (trifluoromethanesulfonyl) imide (Li-TFSI) solution (520 mg Li-TFSI in 1 mL acetonitrile), and 1 mL of chlorobenzene. Cs_2_CO_3_ (0.2 g), ODE (10 mL), OA (0.7 mL) were loaded into a 50 mL flask and degassed for 1 h at 100 ℃ and then heated to 150 ℃ under N_2_ atmosphere until the Cs_2_CO_3_ was completely dissolved.

***Quantum dots Preparation:*** CsPbBr_3_ QDs and CsPbI_3_ QDs were synthesized through a reported method with minor modifications [S1]. PbX_2_ (0.43 mmol) such as PbBr_2_ (0.157g) or PbI_2_ (0.2 g), ODE (10 mL), OA (1 mL) and OAm (1 mL) were loaded into a 50 mL flask and degassed for 1 h at 100 ℃ and then heated to 150 ℃ under N_2_ atmosphere to form a clear solution. The temperature was increased to 170 ℃, followed by the quick injection of 0.8 mL Cs-OA solution. 5 seconds after the injection, the reaction was stopped with an ice bath. The ethyl acetate was added to the crude solution (Ethyl acetate: crude solution=1:1 by volume) to precipitate QDs, then the mixture solution was centrifugation at 7000 rpm for 3 min. The collected CsPbX_3_ QDs were dispersed in toluene to form a solution with 15 mg/mL concentration.

***Device Fabrication:*** The ITO glass substrates were initially cleaned with a detergent, followed by ultrasonic cleaning in deionized water, acetone, and isopropanol for 30 minutes each. The ITO glass was dried with nitrogen gas before undergoing a 5-minute plasma treatment. Subsequently, SnO_2_ was spin-coated onto the substrates at a speed of 3500 rpm to serve as the electron transport layer, followed by thermal annealing at 150°C for 30 minutes on a hot plate. After cooling to room temperature, the substrates were transferred to a nitrogen-filled glovebox. A 1.5M PbI_2_ solution (in anhydrous DMF: DMSO at a volume ratio of 9:1) was then spin-coated onto the SnO_2_ layer at 1700 rpm, annealed at 70°C for 1 minute, and allowed to cool for 10 minutes. Quantum dot solution, at a concentration of 3 mg/mL, was then spin-coated on the PbI_2_ layer, followed by a 70°C anneal for 1 minute. Afterwards, a FAI:MACl solution (90 mg:15 mg in 1 mL IPA) was spin-coated on the PbI_2_ layer at 1800 rpm for 30 seconds, after which the substrates were transferred to an air atmosphere glovebox (RH 30%-40%) for thermal annealing at 150°C for 10 minutes on a hot plate. Post-annealing, the substrates were cooled to room temperature in a nitrogen-filled glovebox. Next, a Spiro-OMeTAD solution was spin-coated onto the prepared perovskite film at 4000 rpm for 30 seconds. Lastly, a gold electrode approximately 80 nm thick was thermally evaporated under high vacuum through a mask.

***Characterizations:*** The crystal structure and phases of the perovskite were characterized using a Bruker Advanced D8 X-ray diffractometer under Cu Kα (λ = 0.154 nm) radiation. The absorbance spectra of the perovskite film were obtained using a UV-Vis spectrophotometer (Agilent Cary 5000). Steady-state photoluminescence (PL) spectra were recorded on a Shimadzu RF-5301pc, while time-resolved photoluminescence spectra were acquired using a picosecond pulsed laser excitation with a 1 MHz repetition rate through a photoluminescence system (Fluo-Time 300). The morphology of the film was investigated using a scanning electron microscope (SEM; TESCAN MIRA3). The surface potential of the perovskite film was measured using an atomic force microscope (AFM; Asylum Research MFP-3D-Stand Alone). X-ray photoelectron spectroscopy (XPS) was performed on a Thermo K-Alpha+ spectrometer equipped with a monochromatic Al Kα X-ray source (1486.6 eV) operating at 100 W power. The samples were analyzed under vacuum (P < 10^-8^ mbar), calibrated through 150 eV (survey scan) or 50 eV (high-resolution scan) energy pass, with a binding energy of C 1s at 284.8 eV for calibration. Ultraviolet photoelectron spectroscopy (UPS, ESCALAB 250Xi, Thermo Fisher) measurements were carried out using a He Iα photon source (21.22 eV). Current-voltage (*J-V*) curves for fabricated devices were obtained by collecting data under forward and reverse scans with 30 mV intervals and a 10 ms delay time, under AM 1.5 G illumination (100 mW cm^-2^) using a source meter (Keysight B2901A) and a solar simulator (Enlitech SS-F5-3A). The external quantum efficiency (EQE) spectra were measured using an Enlitech QER-3011 quantum efficiency test system, calibrated for each wavelength's light intensity using a Si detector before measurement. The maximum power point (MPP) output was obtained from the maximum power point current density. Electrochemical impedance spectroscopy (EIS) tests were conducted using a Princeton Applied Research P4000+ electrochemical workstation in the frequency range from 100 Hz to 1 MHz, at a bias voltage of 1 V under dark conditions with an amplitude of 10 mV.

**S2 Supplementary Figures and Tables**


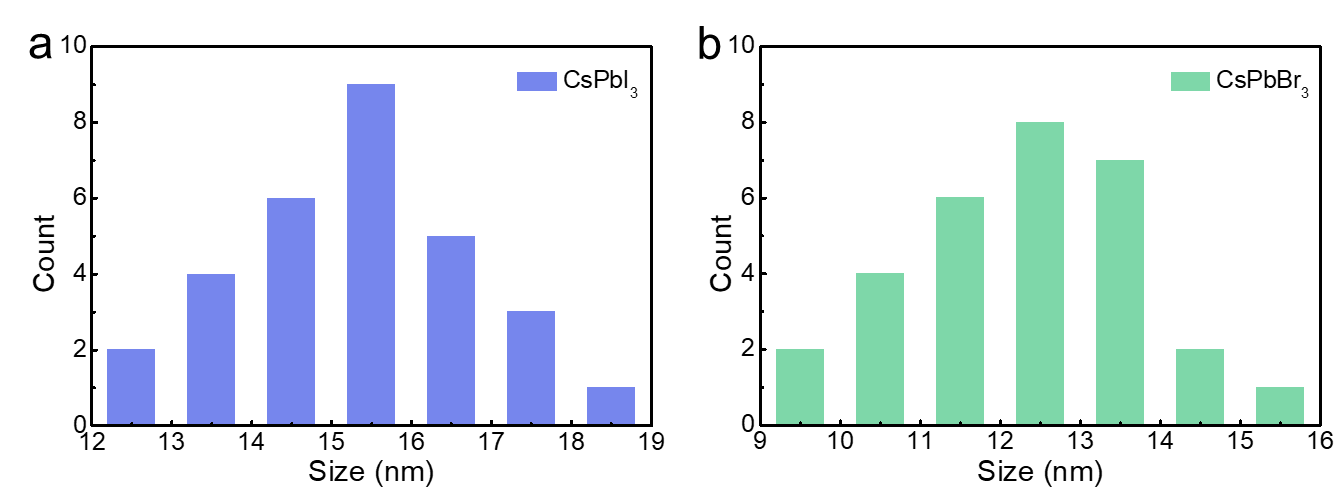


**Fig. S1** Size distribution histograms of CsPbI_3_ and CsPbBr_3_ quantum dots


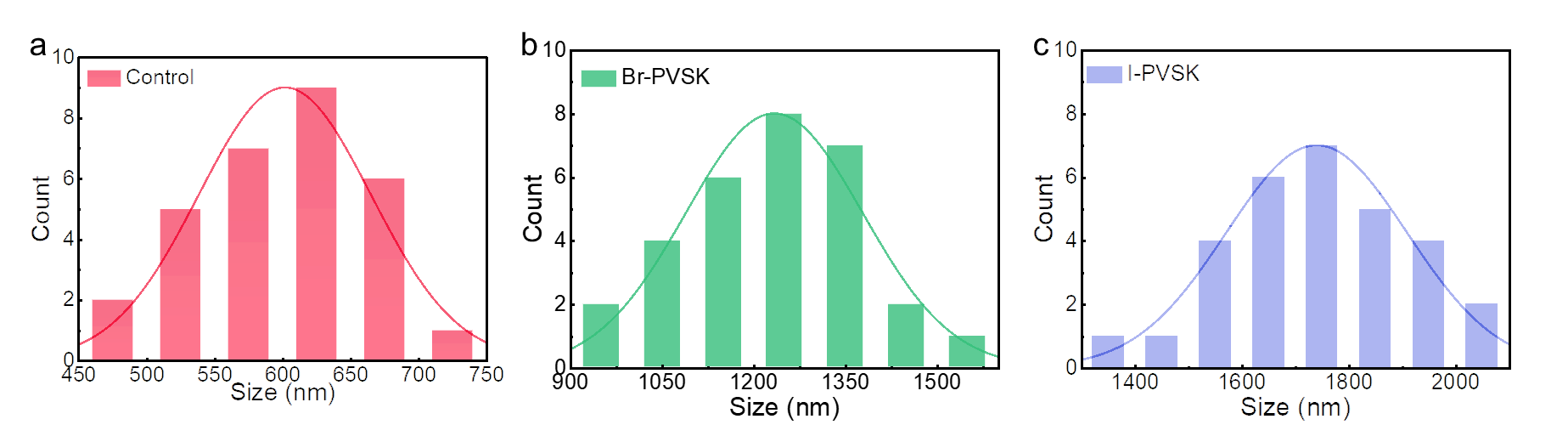


**Fig. S2** (**a-c**) Grain size statistics for Control, Br-PVSK and I-PVSK perovskite films


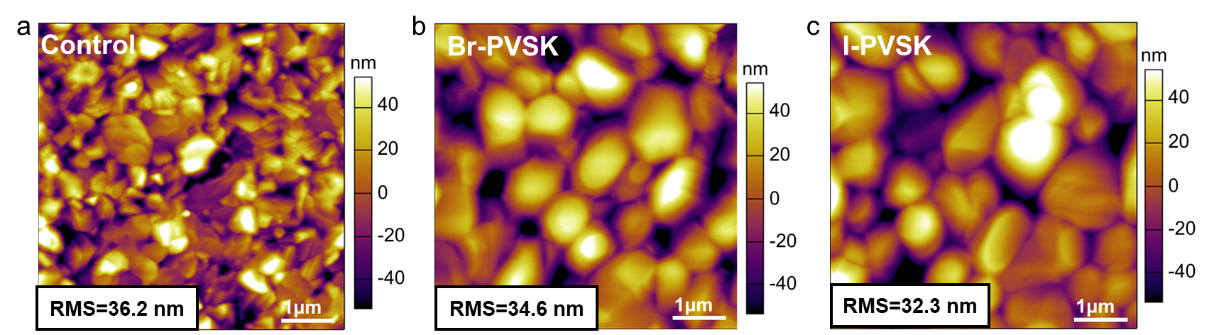


**Fig. S3** AFM topographical images of Control, Br-PVSK and I-PVSK perovskites films


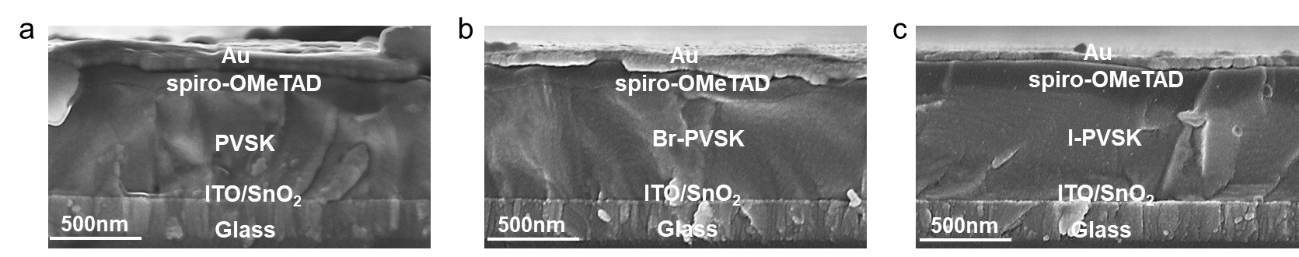


**Fig. S4** (**a-c**) Cross-sectional SEM images of PSC devices based on Control and Br-PVSK, I-PVSK modified


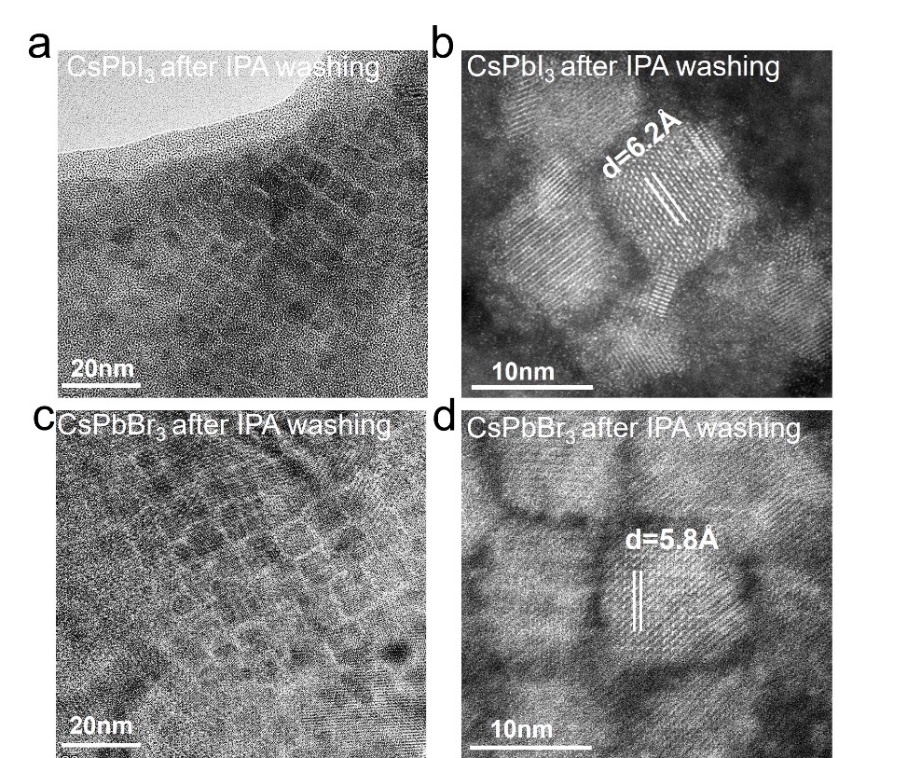


**Fig. S5** TEM morphology of (**a, b**) CsPbI_3_ and (**c, d**) CsPbBr_3_ QDs after IPA washing


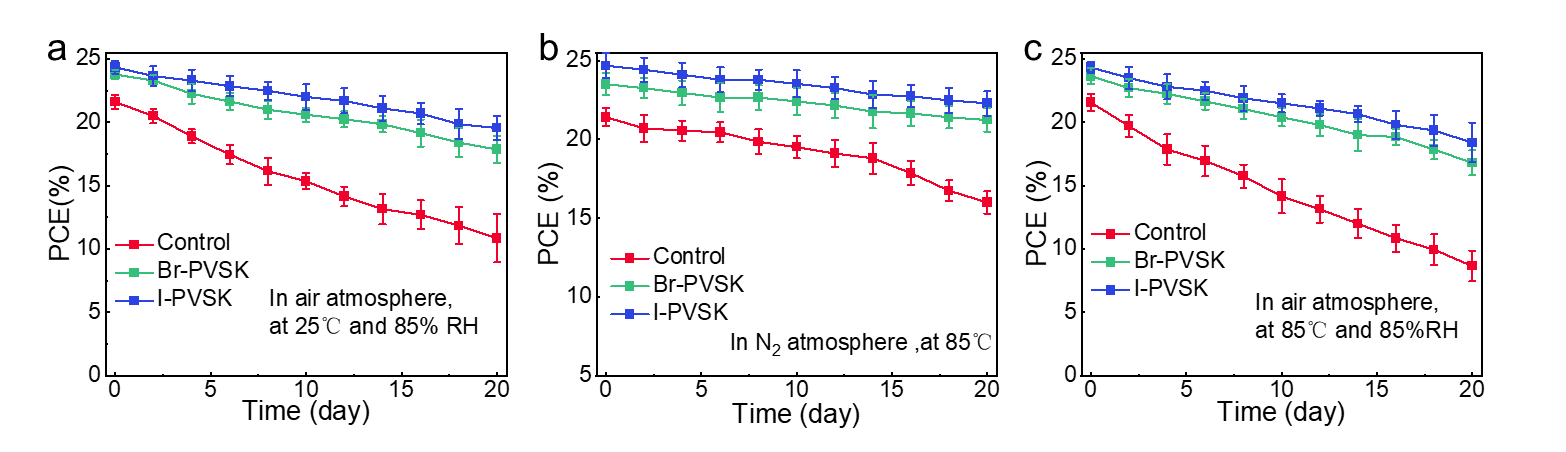


**Fig. S6** (**a**) The humidity stability of unencapsulated PSCs was evaluated in ambient air at approximately 85% relative humidity (RH) at room temperature. The devices were subjected to periodic testing under AM 1.5G illumination using a xenon arc lamp at a power density of 100 mW cm^−2^. (**b**) The thermal stability of unencapsulated PSCs was assessed under continuous heating at 85 °C in a nitrogen atmosphere. These devices were also periodic testing under AM 1.5G irradiation from a xenon arc lamp at 100 mW cm^−2^. (**c**) The stability of unencapsulated PSCs was examined during storage in ambient air at around 85% RH, coupled with heating at 85 °C. Periodic testing of these devices was conducted under AM 1.5G illumination from a xenon arc lamp at a power density of 100 mW cm^−2^


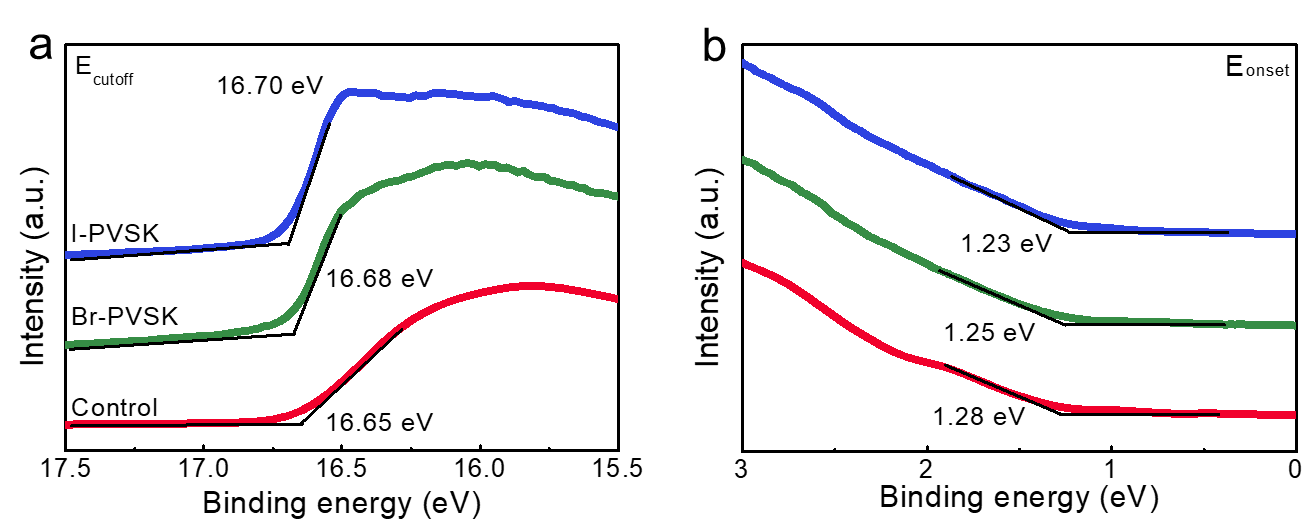


**Fig. S7** UPS spectra of secondary electron cutoff and valence bands for Control, Br-PVSK and I-PVSK perovskite films, respectively


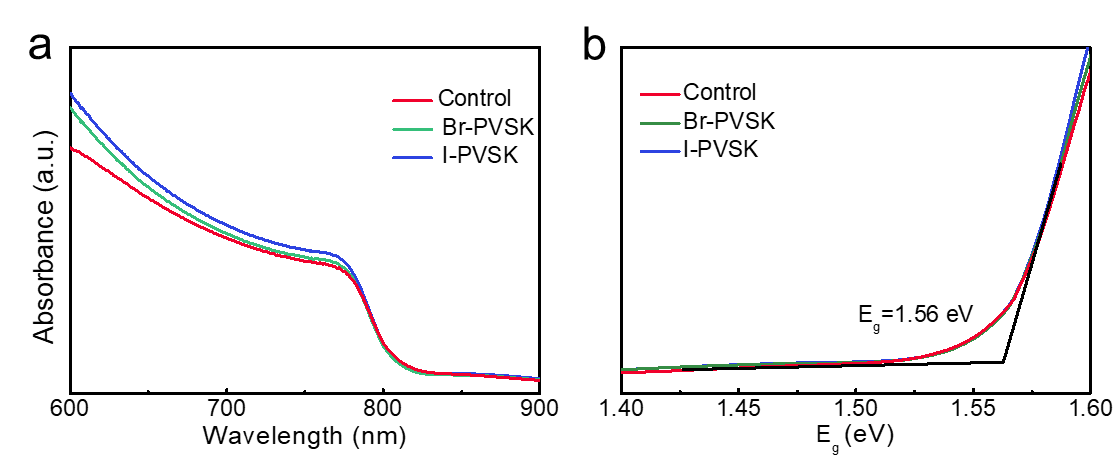


**Fig. S8** (**a**) UV–vis absorption spectra of perovskite films on top of Control, Br-PVSK and I-PVSK. (**b**) Tauc’s plot calculated from the UV-Vis absorption spectra with equation (αhυ)^2^ = A(hυ-E_g_), where α is the absorption coefficient, hυ is the photon energy, and E_g_ is the bandgap


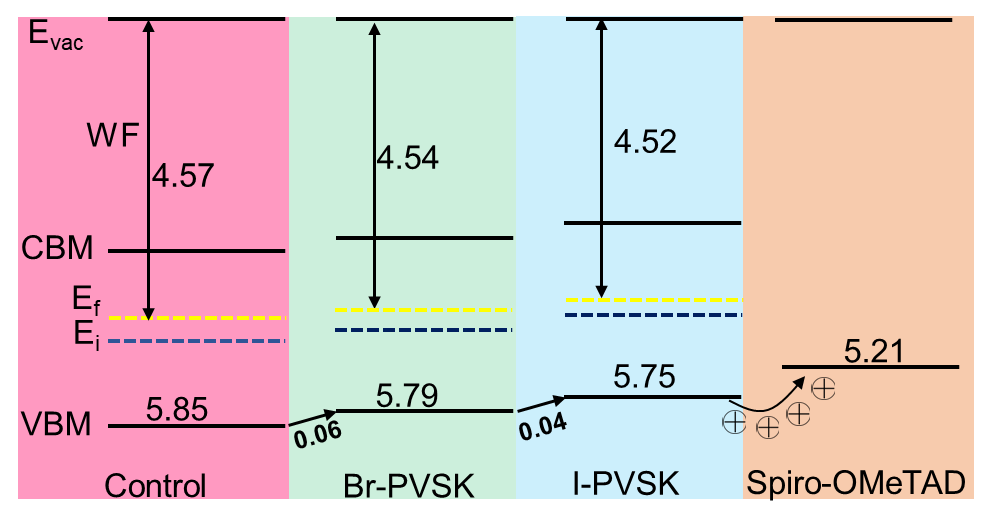


**Fig. S9** Perovskite energy level diagram constructed from UPS results, where E_vac_ stands for vacuum energy level, CBM is conduction band minimum, E_f_ is fermi level of as-deposited perovskite film, E_i_ is fermi level of intrinsic perovskite film, and VBM is valence band maximum


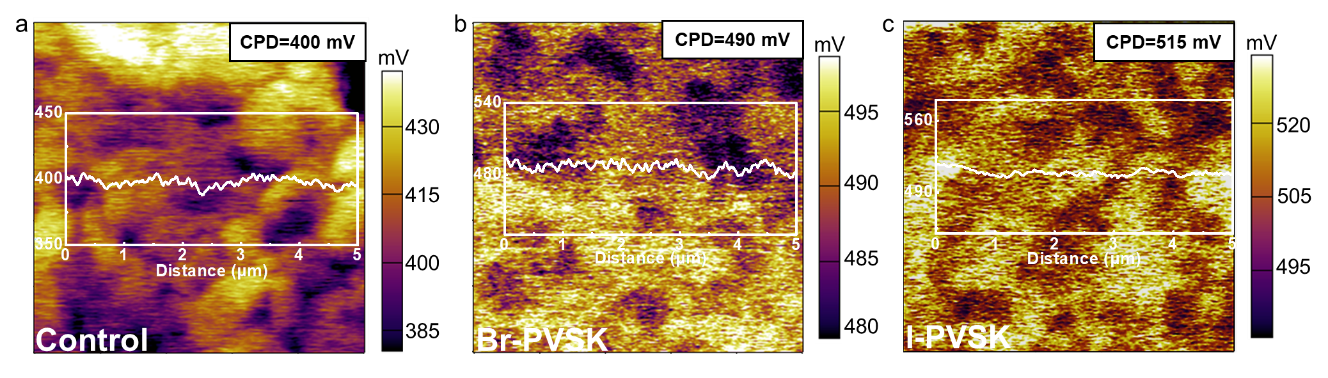


**Fig. S10** KPFM images of Control, Br-PVSK and I-PVSK perovskites films


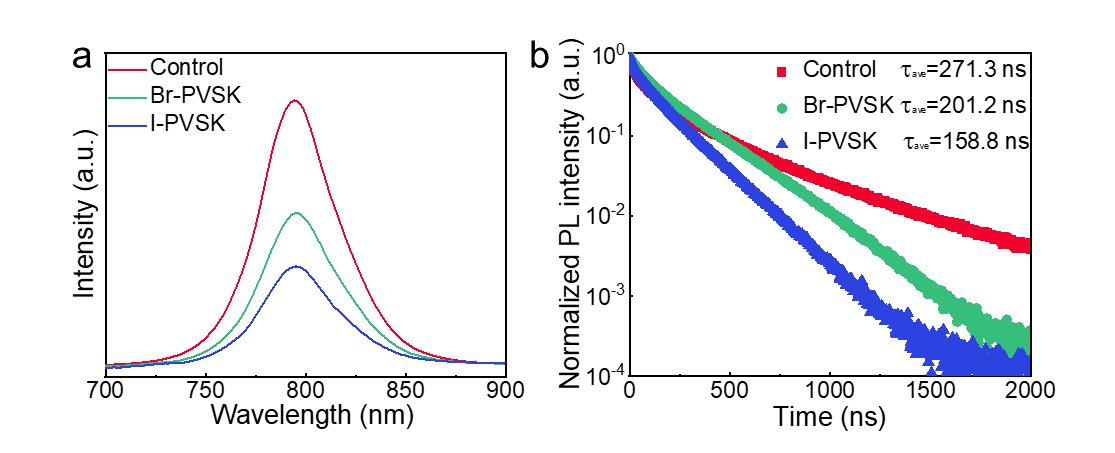


**Fig. S11** (**a**) Steady-state PL and (**b**) TRPL spectra of Glass/PVSK/ Spiro-OMeTAD, Glass/Br-PVSK/Spiro-OMeTAD and Glass/I-PVSK/Spiro-OMeTAD


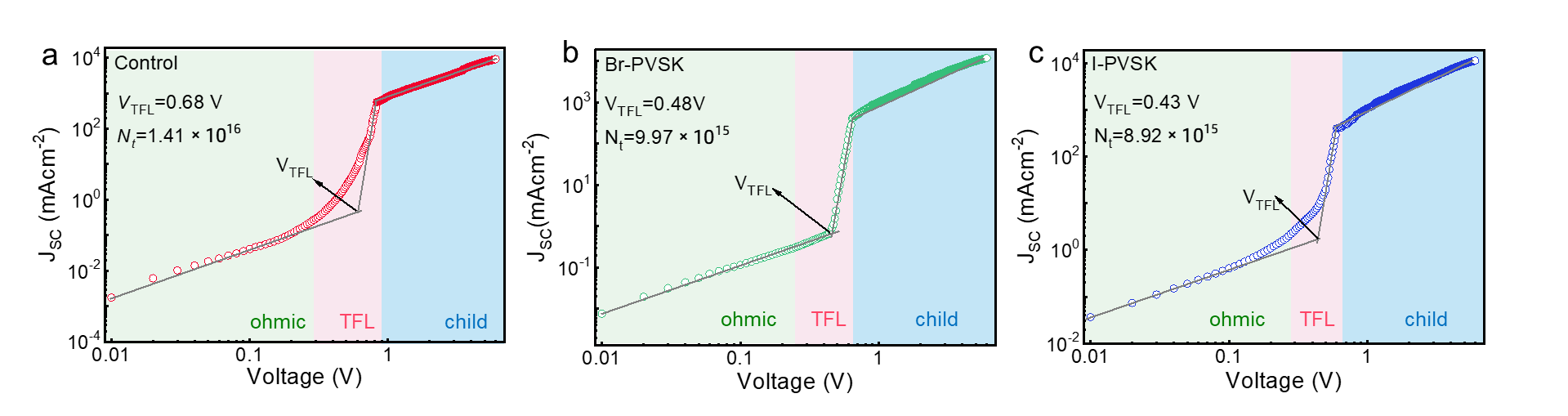


**Fig. S12** (**a−c**) Dark J−V curves representing electron-only devices fabricated by using control, Br-PVSK, and I-PVSK perovskite films

**Table S1** TRPL parameters of the control, Br-PVSK and I-PVSK perovskite films

| Sample | A_1_ | τ_1_ [ns] | A_2_ | τ_2_ [ns] | τ_ave_ [ns] |
| --- | --- | --- | --- | --- | --- |
| Control | 0.51 | 74.91 | 0.53 | 447.40 | 395.60 |
| Br-PVSK | 0.29 | 1346.10 | 1.72 | 267.35 | 759.90 |
| I-PVSK | 0.28 | 1388.40 | 0.79 | 265.90 | 994.50 |

**Table S2** TRPL parameters of the Glass/PVSK/Spiro-OMeTAD, Glass/Br-PVSK/Spiro-OMeTAD and Glass/I-PVSK/Spiro-OMeTAD

| Sample | A_1_ | τ_1_ [ns] | A_2_ | τ_2_ [ns] | τ_ave_ [ns] |
| --- | --- | --- | --- | --- | --- |
| Control | 0.85 | 43.20 | 0.45 | 324.80 | 271.30 |
| Br-PVSK | 0.36 | 55.50 | 0.63 | 205.10 | 201.20 |
| I-PVSK | 0.31 | 32.40 | 0.68 | 169.60 | 158.80 |

**Table S3** Photovoltaic performance of the champion devices for the corresponding PSCs

| Sample | Scan direction | V_oc_ [V] | J_sc_ [mA cm^-2^] | FF [%] | PCE [%] | Integrated J_sc_ [mA cm^–2^] |
| --- | --- | --- | --- | --- | --- | --- |
|  | Forward | 1.106 | 25.26 | 76.64 | 21.43 |  |
| Control | Reverse | 1.116 | 25.34 | 77.04 | 22.05 | 24.20 |
|  | Forward | 1.181 | 25.47 | 79.51 | 23.91 |  |
| Br-PVSK | Reverse | 1.184 | 25.55 | 79.70 | 24.11 | 24.51 |
|  | Forward | 1.194 | 25.38 | 81.42 | 24.67 |  |
| I-PVSK | Reverse | 1.196 | 25.38 | 81.55 | 24.75 | 24.34 |

**Table S4** Conduction band minimum (CBM) and valence band maximum (VBM) of control, Br-PVSK and I-PVSK perovskite films

| Sample | VBM | CBM | E_g_ | WF |
| --- | --- | --- | --- | --- |
| Control | 5.85 | 4.29 | 1.56 | 4.57 |
| Br-PVSK | 5.79 | 4.23 | 1.56 | 4.54 |
| I-PVSK | 5.75 | 4.19 | 1.56 | 4.52 |

**Supplementary Reference**

[S1] L. Protesescu, S. Yakunin, M.I. Bodnarchuk, et al. Nanocrystals of cesium lead halide perovskites (CsPbX_3_, X = Cl, Br, and I): novel optoelectronic materials showing bright emission with wide color gamut. Nano Lett. **15**(6), 3692-3696 (2015). <https://doi.org/10.1021/nl5048779>
